# Supplementary material for: Role of laterodorsal tegmentum projections to nucleus accumbens in reward-related behaviors
Source: Nat Commun. 2019 Sep 12;10:4138. doi: 10.1038/s41467-019-11557-3 (PMC6742663; doi:10.1038/s41467-019-11557-3)
Supplement: Supplementary file 1 — Supplementary Information [file 41467_2019_11557_MOESM1_ESM.pdf]

Supplementary material – Coimbra B. *et al*

Role of laterodorsal tegmentum projections to nucleus accumbens in reward-related behaviors

Bárbara Coimbra, Carina Soares-Cunha, Nivaldo Vasconcelos, Ana Verónica Domingues, Sónia Borges, Nuno Sousa, Ana João Rodrigues

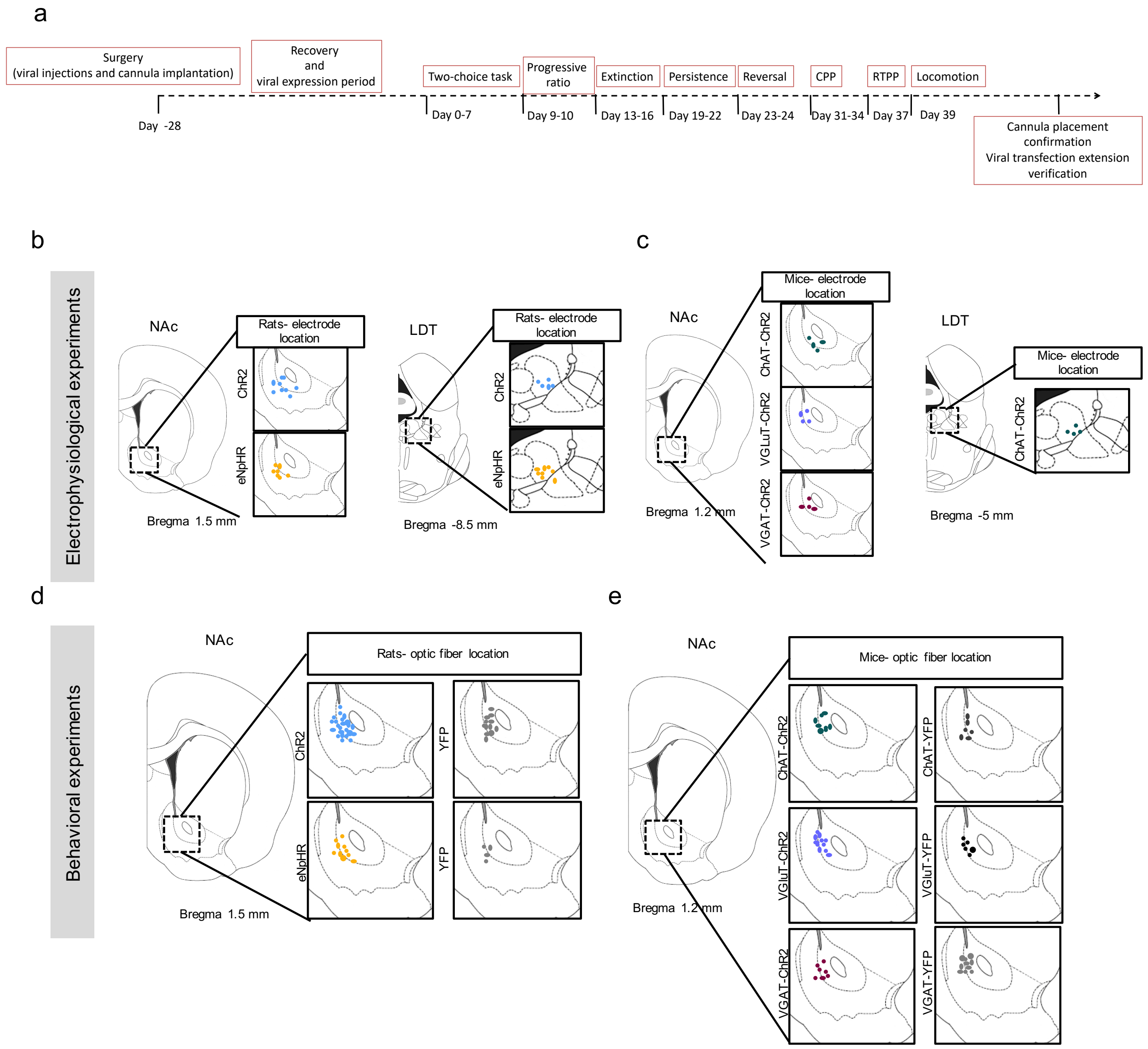

Supplementary Figure 1. Timeline of behavior and electrode and optic fiber placement confirmation. (**a**) Experimental timeline of experiments. (**b-c**) Electrode placement of animals used for electrophysiological experiments: (**b**) rats and (**c**) mice. Schematic representation of optic fiber placement in LDT terminals in the NAc of (**d**) rats and (**e**) mice that performed behavioral experiments.

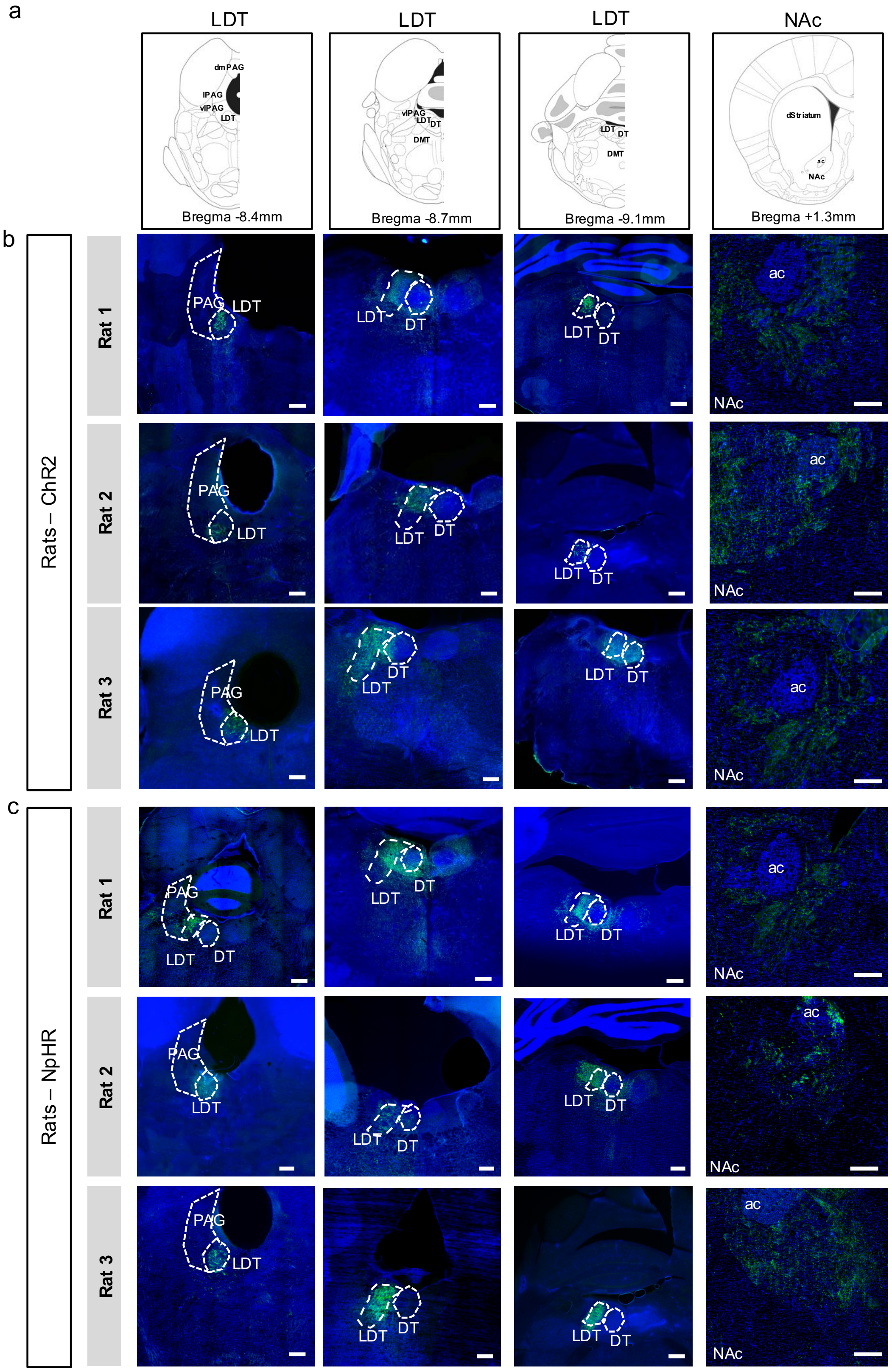

Supplementary Figure 2. Confirmation of viral expression in rats. (**a**) Schematic representation of LDT and NAc coordinates of brain sections depicted in b-c. (**b-c**) Immunofluorescence showing YFP staining in the LDT region of 3 representative animals of ChR2 and NpHR groups; 3 different sections are shown. In the right, it is also depicted one section of the NAc showing the presence of YFP^{+} terminals.

Scale bars from LDT slices=1 mm; scale bars from NAc slice =500 μm PAG: periaqueductal gray; DT: dorsal tegmental nucleus.

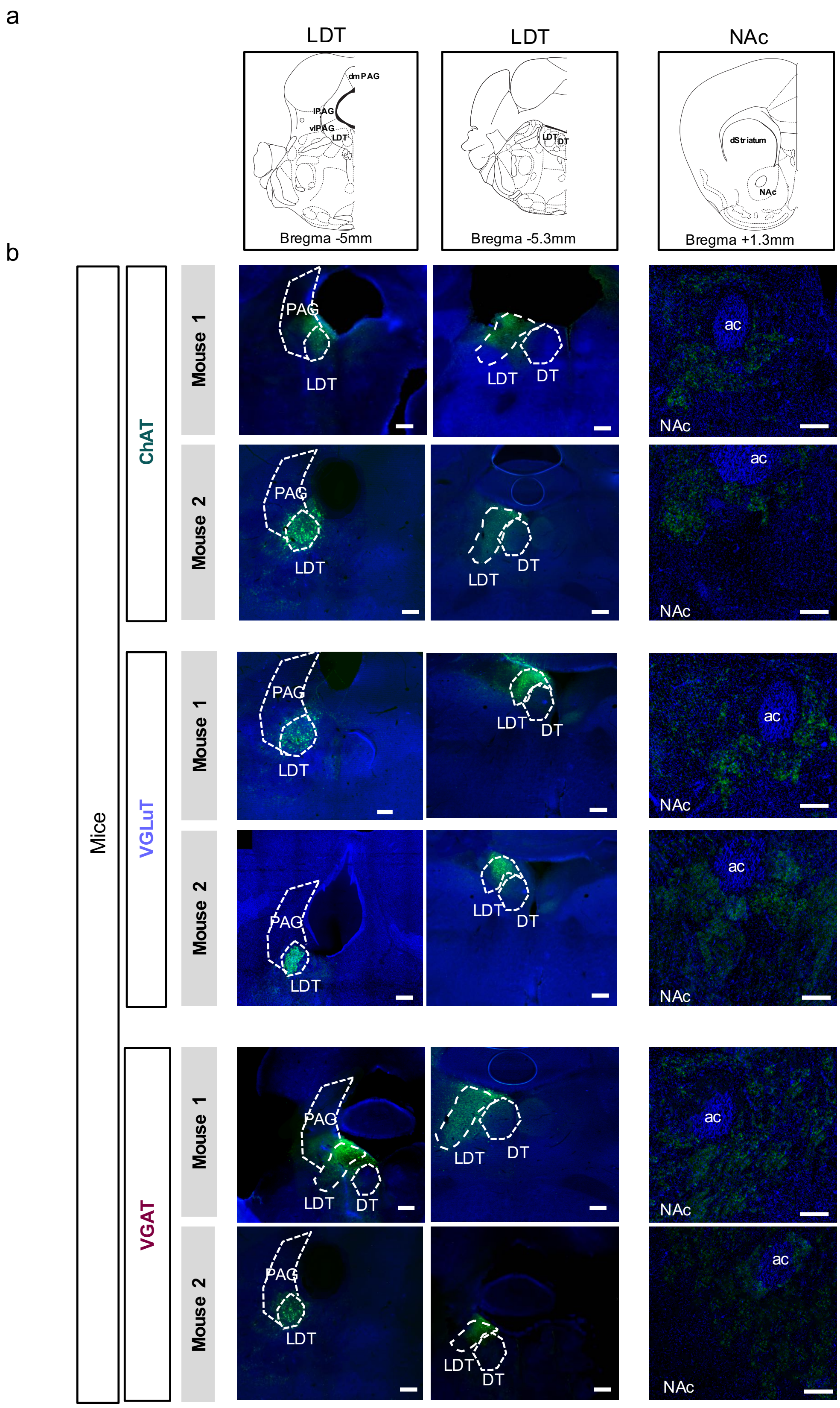

Supplementary Figure 3. Confirmation of viral expression in mice. (**a**) Schematic representation of LDT and NAc coordinates of brain sections depicted in b. (**b**) Immunofluorescence showing YFP staining in the LDT and LDT terminals in the NAc of 2 representative animals of ChAT, VGluT and VGAT groups.

Scale bars from LDT slices =1 mm; scale bars from NAc slice =500 μm; PAG: periaqueductal gray; DT: dorsal tegmental nucleus.

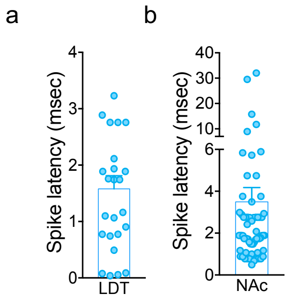

Supplementary Figure 4. Spike latency to optical stimulation. (**a**) Latency of LDT neurons response to soma optical stimulation (80 10ms pulses at 20 Hz). (**b**) Latency of response of NAc neurons to LDT terminal optical stimulation (80 10ms pulses at 20 Hz).

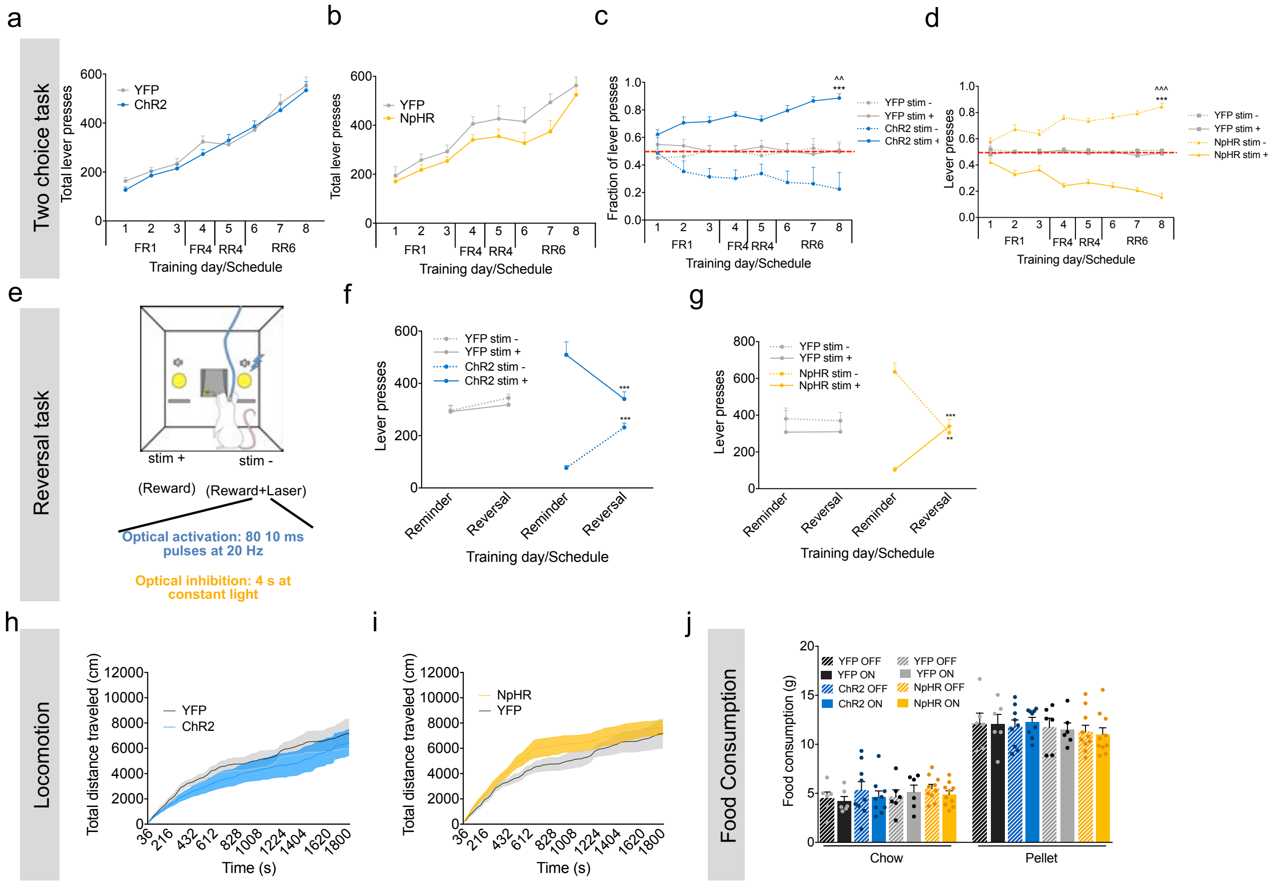

Supplementary Figure 5. Optogenetic modulation of LDT-NAc terminals does not affect behavioral flexibility, food consumption or locomotion. Total number of lever presses in the two-choice task of (**a**) ChR2 and (**b**) NpHR animals are similar to YFP animals. Fraction of lever presses for each session during the two-choice task of (**c**) ChR2 and (**d**) NpHR animals. (**e**) Two-choice task representation of reversal session. (**f**) Reversal session of ChR2 and respective YFP control animals. Stim- was switched for stim+ and vice versa. ChR2 animals shift preference for the new stim+ lever, showing behavioral flexibility. (**g**) Reversal session of NpHR and respective YFP control animals. Stim- was switched for stim+ and vice versa. NpHR animals shift preference for the new stim- lever, showing behavioral flexibility. (**h-i**) No impact in locomotion by optical activation or inhibition of LDT-NAc projections. (**j**) No differences in food consumption of chow or palatable food by optical activation or inhibition of LDT-NAc projections.

*Values are shown as mean ± s.e.m. *refers to difference between ChR2/NpHR stim+ and stim- lever; RM 2way ANOVA. ^refers to difference between ChR2/NpHR stim+ and YFP stim+ lever; RM 2way ANOVA. **p<0.01, ***p<0.001.*

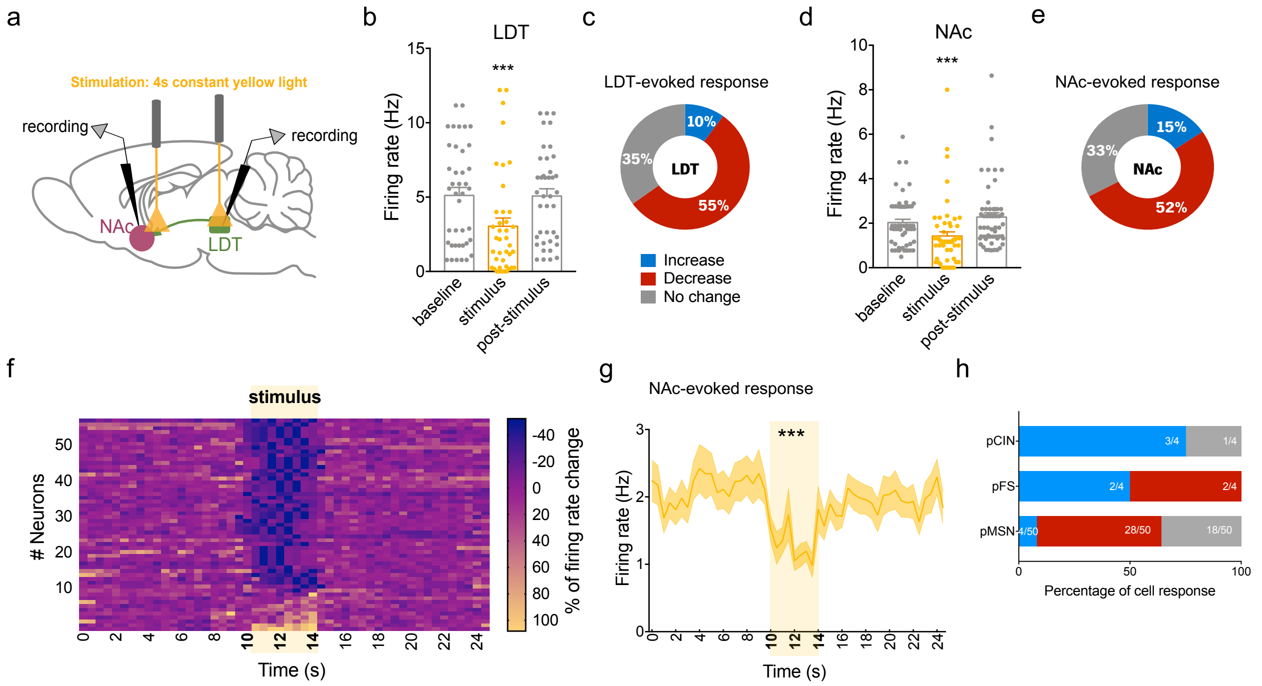

Supplementary Figure 6. LDT-NAc terminal optical inhibition decreases NAc electrophysiological activity. (**a**) Strategy used for optogenetic inhibition and electrophysiological recordings. An AAV-WGA-cre fusion vector was injected unilaterally in the NAc and a cre-dependent NpHR in the LDT. (**b**) During optical inhibition (4s of constant yellow light at 10mW) of LDT cell bodies, LDT decreases firing rate (n=8 animals; 40 LDT cells; RM 1way ANOVA). (**c**) 55% of LDT recorded cells decreased their firing rate during inhibition while no change was detected in 35% of cells; 10% increased the firing rate. (**d**) Firing rate in the NAc is decreased during optical inhibition of LDT terminals (n=7 animals; 58 cells; RM 1way ANOVA). (**e**) Around half of recorded cells in the NAc showed decreased the firing rate upon stimulation, 33% presented no change and 15% increased activity. (**f**) Heatmap representation of cell responses in the NAc to stimulation of LDT terminals with yellow laser. (**g**) Average firing rate of NAc neurons showing a decrease from the baseline during stimulation of LDT-NAc inputs (KS test). (**h**) During LDT terminal inhibition, 56% of recorded MSNs decreased their activity (28/50 cells), 36% did not change firing rate (18/50 cells) and 8% increased their activity (4/50 cells); 75% pCINs (3/4 cells) and 50% pFS (2/4 cells) interneurons increased, 25% pCINs (1/4 cells) did not change and 50% pFS (2/4 cells) interneurons decreased their activity.

*Values are shown as mean ± s.e.m. ***p<0.001.*

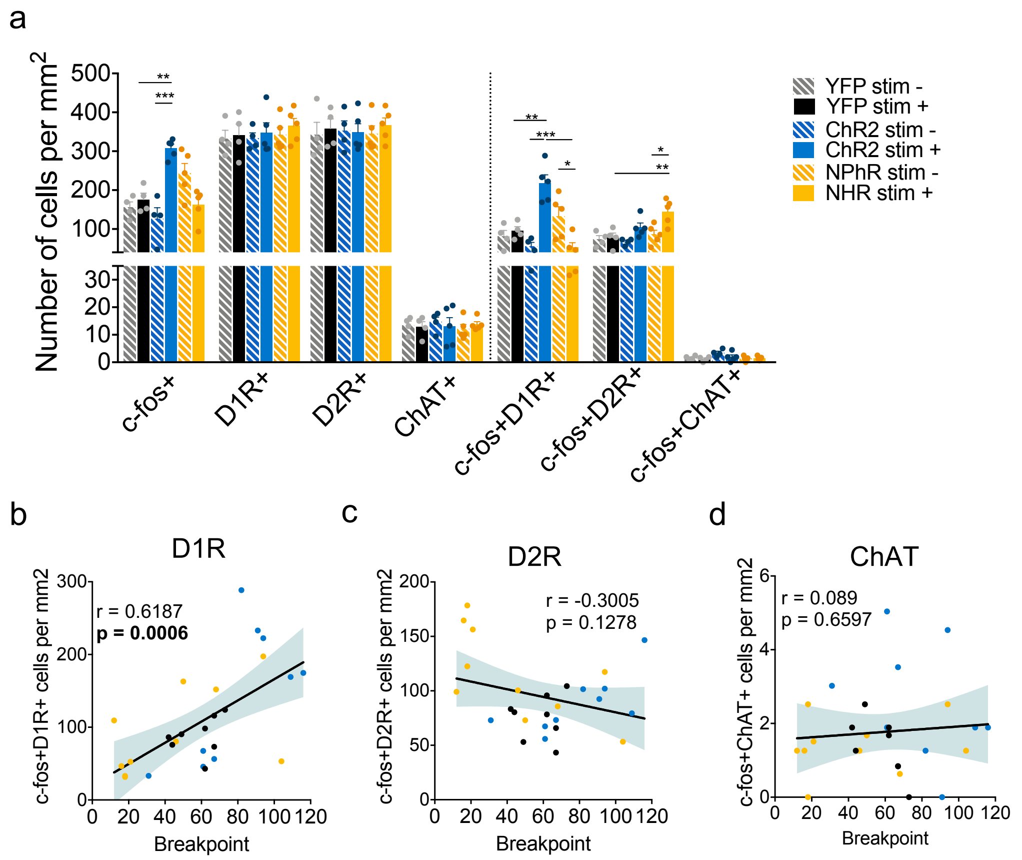

Supplementary Figure 7. Optical activation of LDT-NAc terminals mainly recruits NAc D1-MSNs. (**a**) Quantification of c-fos and D1R, DR2 or ChAT double positive cells in ChR2 (n_{stim+}=5; n_{stim-}=4), NpHR (n_{stim+}=5; n_{stim-}=5) and YFP (n_{stim+}=4; n_{stim-}=4) rats after PR performance on the stim+ or stim- lever. There is a substantial increase in the number of c-fos^{+}/D1R^{+} cells after LDT-NAc optical activation, whereas optical inhibition appears to recruit mostly D2R cells. No significant differences were found in the number of c-fos^{+}/ChAT^{+} cells (1 way ANOVA). (**b**) Pearson's correlation between individual breakpoint of ChR2, NpHR or YFP animals and the number of c-fos^{+}/D1^{+} cells in the NAc. There is a positive correlation between the number of D1R recruited cells in the NAc and individual motivational drive (given by the breakpoint in the PR task). (**c**) Pearson's correlation between individual breakpoint and the number of c-fos^{+}/D2^{+} cells. No significant differences were found. (**d**) Pearson's correlation between individual breakpoint and the number of c-fos^{+}/ChAT^{+} cells. No significant differences were found.

*Values are shown as mean ± s.e.m. *p<0.05; **p<0.01; ***p<0.001.*

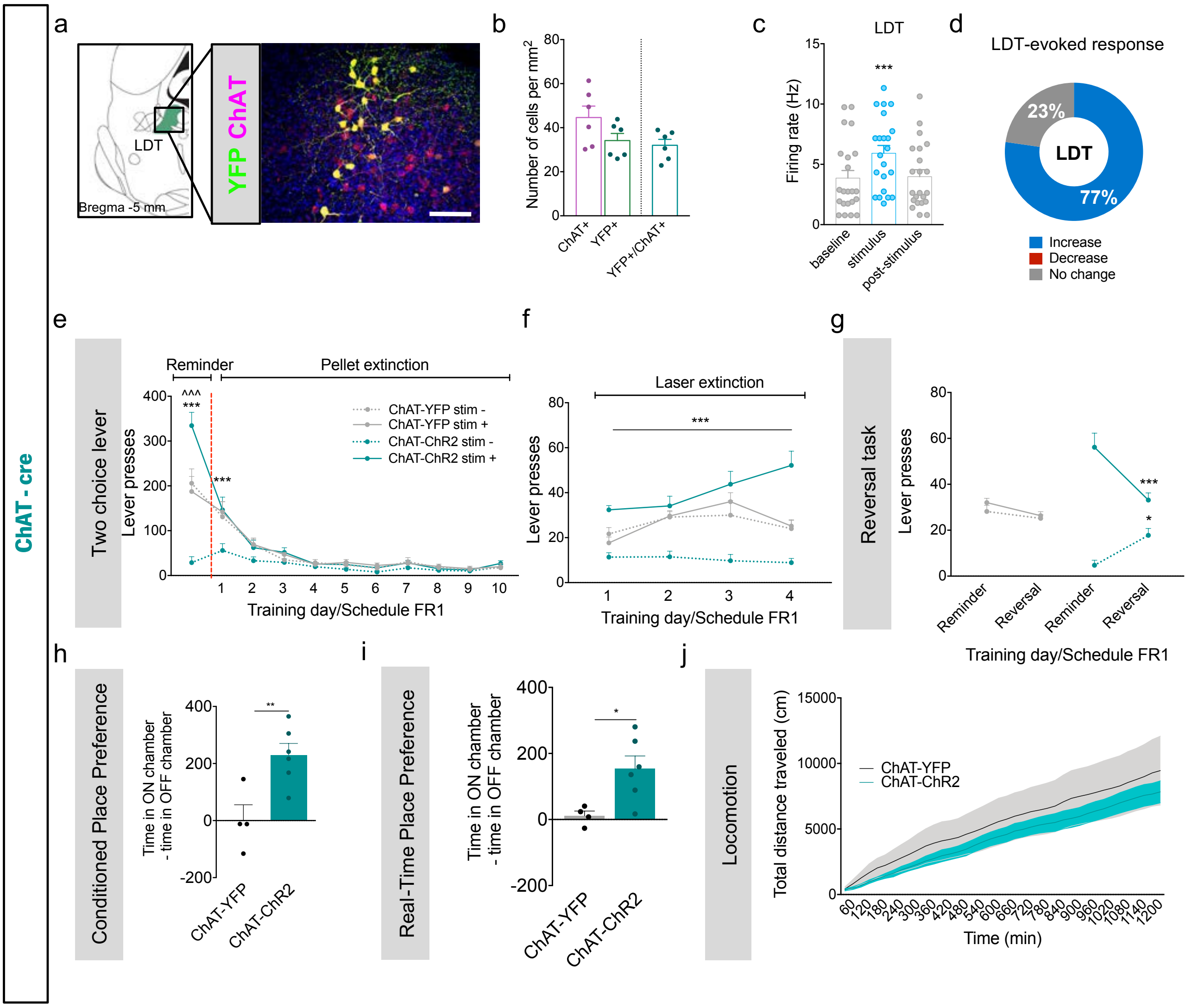

Supplementary Figure 8. Behavioral effects of optical activation of LDT-NAc cholinergic terminals. (**a**) Representative immunofluorescence for YFP and ChAT; scale bar=100 μm. (**b**) Respective quantification of double positive cells (n=6 animals). (**c**) LDT neurons increase firing rate in response to optical activation of LDT cell bodies (stimulation: 80 10 ms pulses at 20 Hz) (n=4 animals; 22 cells; RM 1way ANOVA). (**d**) 77% of LDT recorded cells increased their firing rate to optical stimulation. (**e**) In pellet extinction conditions, ChAT-ChR2 and ChAT-YFP groups decrease responses for stim+ and stim- levers, despite pressing stim+ still originates LDT-NAc cholinergic terminals stimulation. (**f**) In laser extinction conditions, ChAT-ChR2 animals still manifest preference for the stim+ lever, despite no stimulation is given; ChAT-YFP animals do not manifest preference. (**g**) ChAT-ChR2 animals shift their preference for the new stim+ lever in a reversal task. *refers to difference between ChAT-ChR2 stim+ and stim- lever, RM 2way ANOVA; ^refers to difference between ChAT-ChR2 stim+ and ChAT-YFP stim+ lever, RM 2way ANOVA. Difference between time spent in the ON and OFF chambers in the (**h**) CPP and (**i**) RTPP, showing preference for the ON chamber in both tests (t test). (**j**) No significant impact in locomotion by optical activation of LDT-NAc cholinergic projections ($n_{ChAT\text{-}ChR2}$=6, 2 animals lost cannula; $n_{ChAT\text{-}YFP}$=4, 2 animals lost cannula).

*Values are shown as mean ± s.e.m. *p<0.05; **p<0.01; ***p<0.001.*

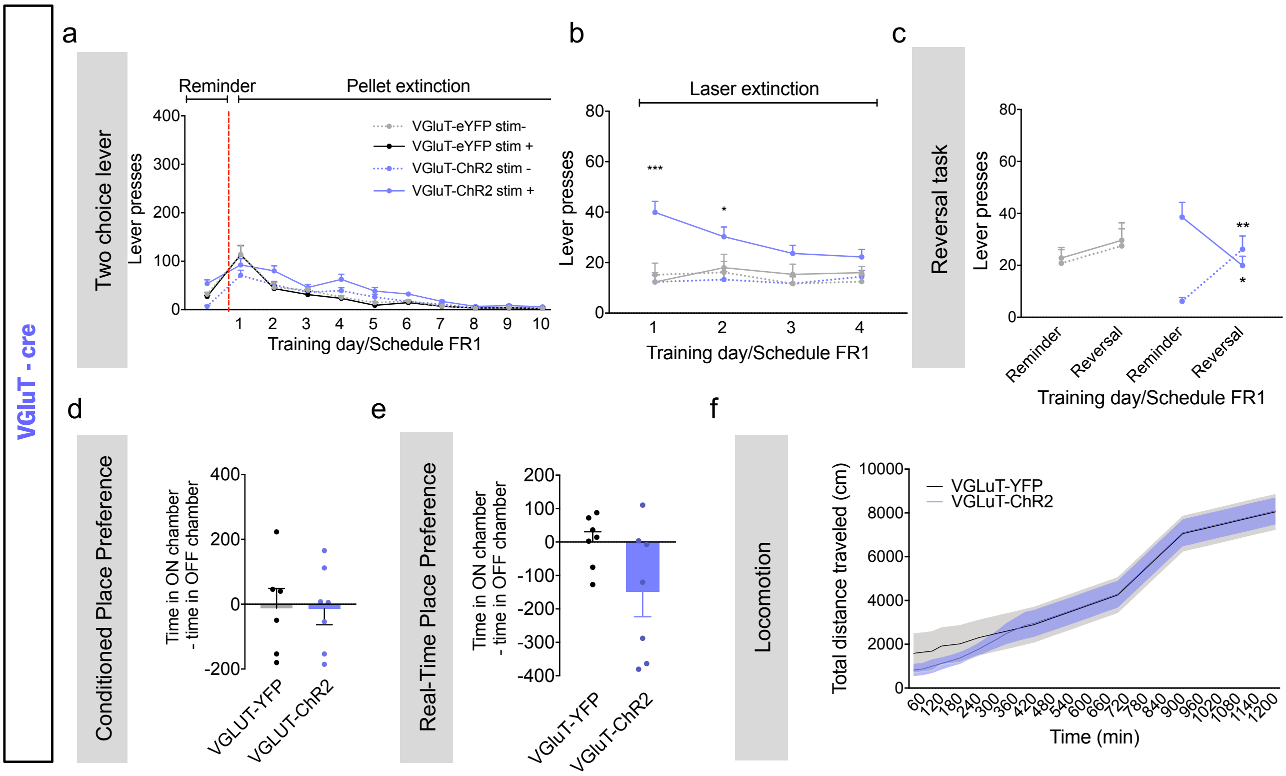

Supplementary Figure 9. Behavioral effects of optical activation of LDT-NAc glutamatergic terminals. (**a**) During pellet extinction conditions, VGluT-ChR2 and VGluT-YFP animals decreased lever pressing for both levers. (**b**) VGluT-ChR2 animals retain preference for stim+ lever in laser extinction conditions for the first 2 sessions, though they decrease the number of presses throughout sessions. (**c**) VGluT-ChR2 animals shift their preference for the new stim+ lever in a reversal task. (**d**) Difference between time spent in the ON and OFF chambers, showing no differences between groups. (**e**) Difference between time spent in the ON and OFF chambers in the RTPP. (**f**) No impact in locomotion by optical activation of LDT-NAc glutamatergic projections.

*Values are shown as mean ± s.e.m. *refers to difference between VGluT-ChR2 stim+ and stim- lever, RM 2way ANOVA. *p<0.05; **p<0.01; ***p<0.001.*

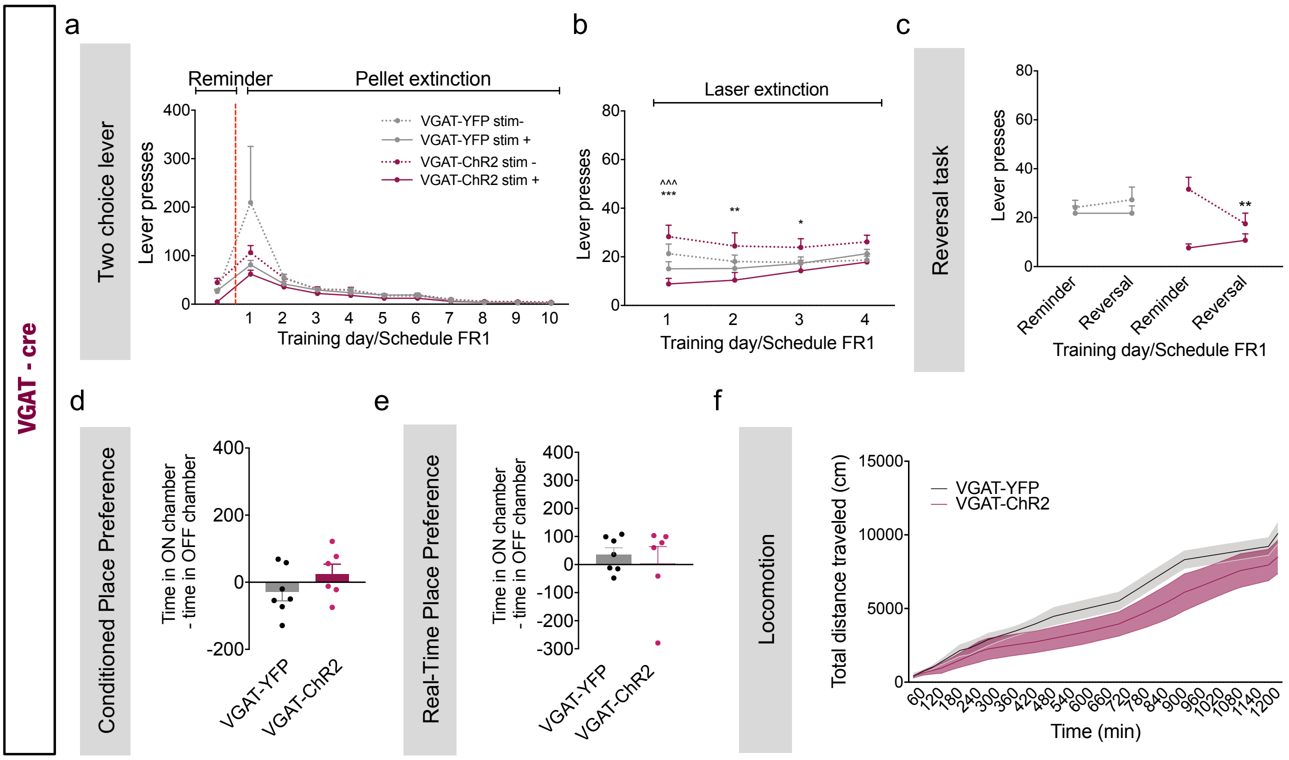

Supplementary Figure 10. Behavioral effects of optical activation of LDT-NAc GABAergic terminals. (**a**) In pellet extinction conditions, VGAT-ChR2 and VGAT-YFP have reduced number of presses in both levers. (**b**) In laser extinction conditions, VGAT-ChR2 animals still retain preference for the stim- lever. (**c**) VGAT-ChR2 animals decrease their preference for the new stim+ lever in a reversal task. (**d**) Difference between time spent in the ON and OFF chambers in the CPP test, showing no differences between groups. (**e**) Difference between time spent in the ON and OFF chambers in the RTPP. (**f**) No impact in locomotion by optical activation of LDT-NAc GABAergic projections ($n_{VGAT\text{-}ChR2}$=6, 1 animal lost cannula; $n_{VGAT\text{-}YFP}$=7, 4 animals lost cannula).

*Values are shown as mean ± s.e.m. *refers to difference between VGAT-ChR2 stim+ and stim- lever, RM 2way ANOVA; ^refers to difference between VGAT-ChR2 stim+ and VGAT-YFP stim+ lever, RM 2way ANOVA. *p<0.05; **p<0.01; ***p<0.001*

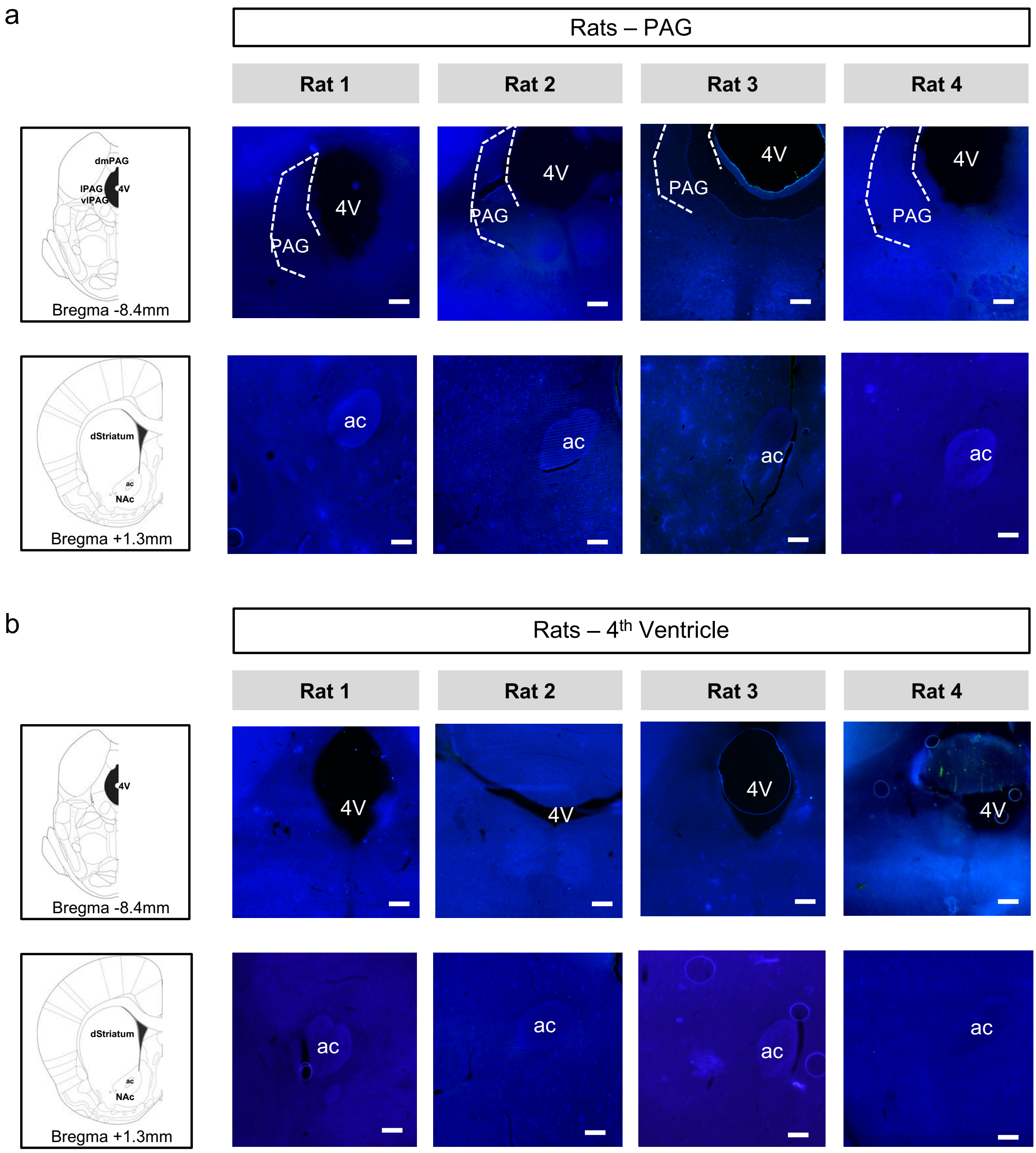

Supplementary Figure 11. Histological evaluation of control groups injected in the periaqueductal grey (PAG) and 4th ventricle (4V). (**a**) One group of animals was injected with WGA-cre in NAc + DIO-ChR2 in the PAG. No YFP expression was found in the PAG nor in the NAc. (**b**) Another group of animals was injected with WGA-cre in NAc + DIO-ChR2 in the 4th Ventricle. No expression of YFP+ cells in the 4th Ventricle nor adjacent areas, nor YFP+ terminals in the NAc was found.

Scale bars = 0.5 mm.

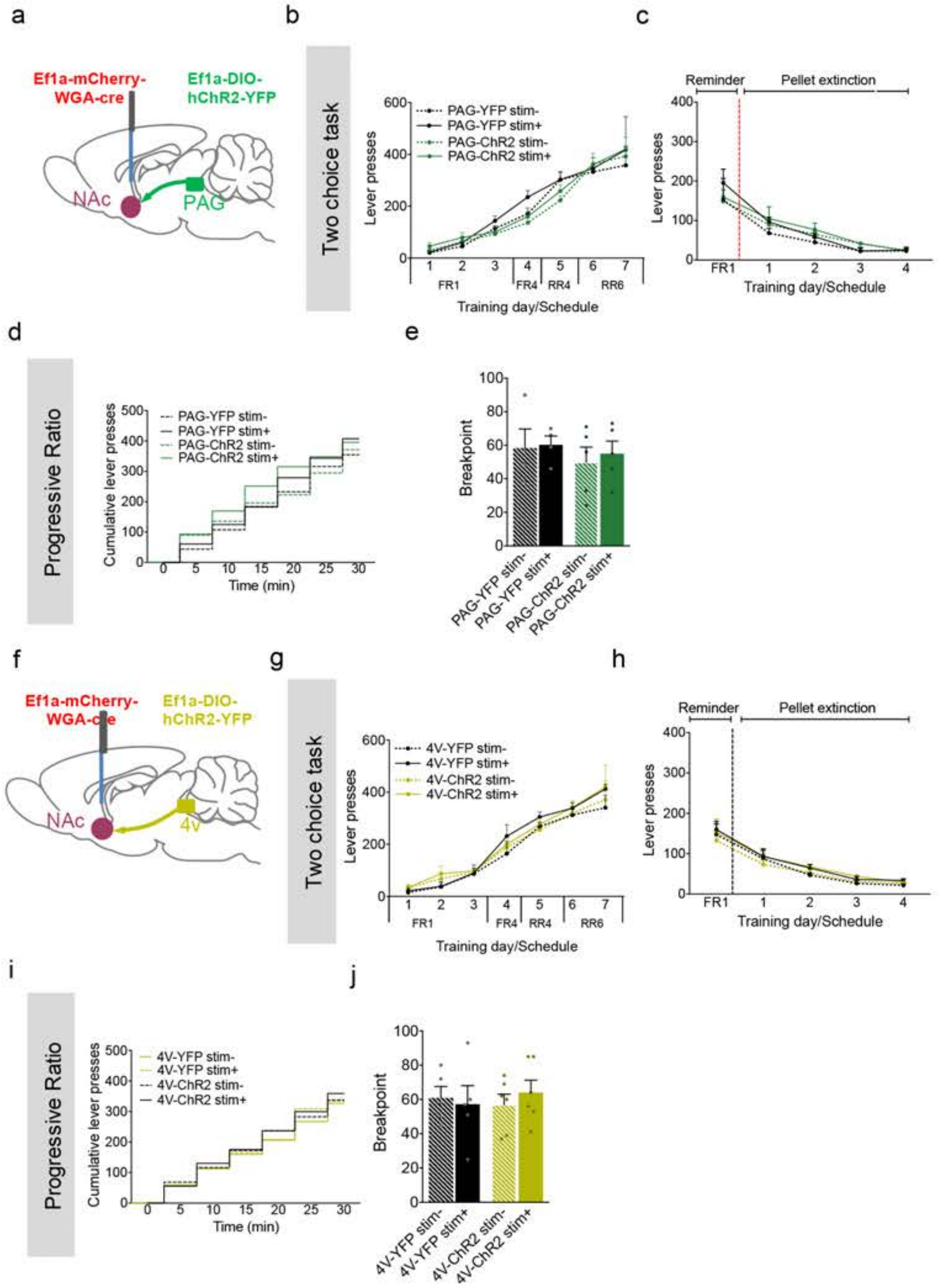

Supplementary Figure 12. Behavioral evaluation of control groups, in which PAG-NAc or 4thV-NAc "terminals" were stimulated (vide also Sup. Fig. 11, showing the absence of staining, indicative of the lack of projections of either region). (**a**) Strategy used in order to stimulate PAG hypothetical terminals in the NAc. One group of animals was injected with WGA-cre in NAc + DIO-ChR2 in the PAG, and optically stimulated in the NAc. (**b**) Stimulation of hypothetic PAG terminals in the NAc did not induce any preference in the two-choice task. (**c**) All groups decrease responding in pellet extinction conditions. (**d-e**) No effect of "PAG-NAc terminals" optogenetic activation in the progressive ratio test ($n_{PAG-ChR2}$=5, $n_{PAG-YFP}$=4). (**f**) Strategy used to control for misplaced injection in the 4V. One group of animals was injected with WGA-cre in NAc + DIO-ChR2 in the 4V. (**g**) Stimulation of hypothetic terminals in the NAc did not induce any preference in the two-choice task. (**h**) All groups decrease responding in pellet extinction conditions. (**i-j**) No effect of optogenetic activation in the progressive ratio test ($n_{4V-ChR2}$=6, n_{4V-YFP}=5).

Values are shown as mean ± s.e.m.
