# Supplementary material for: Role of laterodorsal tegmentum projections to nucleus accumbens in reward-related behaviors
Source: Nat Commun. 2019 Sep 12;10:4138. doi: 10.1038/s41467-019-11557-3 (PMC6742663; doi:10.1038/s41467-019-11557-3)
Supplement: Supplementary file 3 — Reporting Summary [file 41467_2019_11557_MOESM3_ESM.pdf]

nature research

Corresponding author(s): Ana João Rodrigues

Last updated by author(s): Jul 4, 2019

Reporting Summary

Nature Research wishes to improve the reproducibility of the work that we publish. This form provides structure for consistency and transparency in reporting. For further information on Nature Research policies, see Authors & Referees and the Editorial Policy Checklist.

Statistics

For all statistical analyses, confirm that the following items are present in the figure legend, table legend, main text, or Methods section.

n/a	Confirmed	
☐	☒	The exact sample size (n) for each experimental group/condition, given as a discrete number and unit of measurement
☐	☒	A statement on whether measurements were taken from distinct samples or whether the same sample was measured repeatedly
☐	☒	The statistical test(s) used AND whether they are one- or two-sided<br>*Only common tests should be described solely by name; describe more complex techniques in the Methods section.*
☒	☐	A description of all covariates tested
☐	☒	A description of any assumptions or corrections, such as tests of normality and adjustment for multiple comparisons
☐	☒	A full description of the statistical parameters including central tendency (e.g. means) or other basic estimates (e.g. regression coefficient) AND variation (e.g. standard deviation) or associated estimates of uncertainty (e.g. confidence intervals)
☐	☒	For null hypothesis testing, the test statistic (e.g. F, t, r) with confidence intervals, effect sizes, degrees of freedom and P value noted<br>*Give P values as exact values whenever suitable.*
☒	☐	For Bayesian analysis, information on the choice of priors and Markov chain Monte Carlo settings
☒	☐	For hierarchical and complex designs, identification of the appropriate level for tests and full reporting of outcomes
☐	☒	Estimates of effect sizes (e.g. Cohen's d, Pearson's r), indicating how they were calculated

Our web collection on statistics for biologists contains articles on many of the points above.

Software and code

Policy information about availability of computer code

Data collection	*Provide a description of all commercial, open source and custom code used to collect the data in this study, specifying the version used OR state that no software was used.*
Data analysis	GraphPad Prism 5.0 and SPSS Statistics v19.0

For manuscripts utilizing custom algorithms or software that are central to the research but not yet described in published literature, software must be made available to editors/reviewers. We strongly encourage code deposition in a community repository (e.g. GitHub). See the Nature Research guidelines for submitting code & software for further information.

Data

Policy information about availability of data

All manuscripts must include a data availability statement. This statement should provide the following information, where applicable:

- Accession codes, unique identifiers, or web links for publicly available datasets
- A list of figures that have associated raw data
- A description of any restrictions on data availability

The data that support the findings of this study are available from the corresponding author on request.

Field-specific reporting

Please select the one below that is the best fit for your research. If you are not sure, read the appropriate sections before making your selection.

☒ Life sciences ☐ Behavioural & social sciences ☐ Ecological, evolutionary & environmental sciences

Life sciences study design

All studies must disclose on these points even when the disclosure is negative.

Sample size	Sample size is similar to samples sizes routinely used in the field for similar electrophysiological or behavioral comparisons.
Data exclusions	No data was excluded from the study.
Replication	Main behavioral tests were replicated. Electrophysiological recordings were not replicated as they include sufficient number of cells for comparison.
Randomization	Animals were allocated to groups randomly.
Blinding	Investigators were not blinded to mouse genotypes during behavioral experiments, but the data is acquired in an automatic manner. Blinding was used during cell quantification analysis.

Reporting for specific materials, systems and methods

We require information from authors about some types of materials, experimental systems and methods used in many studies. Here, indicate whether each material, system or method listed is relevant to your study. If you are not sure if a list item applies to your research, read the appropriate section before selecting a response.

Materials & experimental systems

n/a	Involved in the study
☐	☒ Antibodies
☒	☐ Eukaryotic cell lines
☒	☐ Palaeontology
☐	☒ Animals and other organisms
☒	☐ Human research participants
☒	☐ Clinical data

Methods

n/a	Involved in the study
☒	☐ ChIP-seq
☒	☐ Flow cytometry
☒	☐ MRI-based neuroimaging

Antibodies

Antibodies used	Primary antibodies used: mouse anti-D2 receptor (1:500, B-10, Santa Cruz Biotechnology); rabbit anti-c-fos (1:1,000, Ab-5, Merck Millipore), goat anti-GFP (1:500, ab6673, Abcam), mouse anti-D1 receptor (1:100, NB110-60017, Novus), rabbit anti GAD65 +GAD67 (1:1000, ab11070, Abcam), mouse anti-EAAC1 (1:500, MAB1587, Millipore), goat anti-ChAT (1:750, AB144P, Millipore) or sheep anti-ChAT (1:500, ab18736, Abcam). Secondary fluorescent antibodies: donkey anti-mouse Alexa 594, donkey anti-goat Alexa 488, donkey anti-rabbit Alexa 594, donkey anti-goat Alexa 647, donkey anti-rabbit Alexa 488 (1:1000, Invitrogen, MA, USA). Antibody specificity was evaluated using the proper negative controls.
Validation	All antibodies were previously used in other studies or were validated by the manufacturing company.

Animals and other organisms

Policy information about studies involving animals; ARRIVE guidelines recommended for reporting animal research

Laboratory animals	Wistar Han rats, aged 2 months at the beginning of experiments. Male and female C57/Bl6 transgenic mice (age of 2 months at the beginning of the experiments) were used: progeny produced by homozygous VGAT-cre (Vgat-IRES-Cre: Slc32a1tm2(cre)Lowl/J, The Jackson Laboratory) and VGluT-cre (VGluT2-IRES-Cre: Slc17a6tm2(cre)Lowl/J, The Jackson Laboratory). The progeny produced by mating ChAT-cre (B6;129S6-Chattm2(cre)Lowl/J/ChAT-IRES-Cre, #006410, The Jackson Laboratory) heterozygous transgenic male mice with wild-type C57/Bl6 females were genotyped at weaning by PCR fragment analysis.
Wild animals	*Provide details on animals observed in or captured in the field; report species, sex and age where possible. Describe how animals were caught and transported and what happened to captive animals after the study (if killed, explain why and describe method; if released, say where and when) OR state that the study did not involve wild animals.*
Field-collected samples	*For laboratory work with field-collected samples, describe all relevant parameters such as housing, maintenance, temperature, photoperiod and end-of-experiment protocol OR state that the study did not involve samples collected from the field.*
Ethics oversight	All manipulations were conducted in strict accordance with European Regulations (European Union Directive 2010/63/EU). Animal facilities and the people directly involved in animal experiments were certified by the Portuguese regulatory entity – DGAV. All the experiments were approved by the Ethics Committee of the University of Minho (SECVS protocol #107/2015). The experiments were also authorized by the national competent entity DGAV (#19074).

Note that full information on the approval of the study protocol must also be provided in the manuscript.
